# Multiple *Klebsiella pneumoniae* KPC Clones Contribute to an Extended Hospital Outbreak

**DOI:** 10.3389/fmicb.2019.02767

**Published:** 2019-11-29

**Authors:** Carolina Ferrari, Marta Corbella, Stefano Gaiarsa, Francesco Comandatore, Erika Scaltriti, Claudio Bandi, Patrizia Cambieri, Piero Marone, Davide Sassera

**Affiliations:** ^1^Microbiology and Virology Unit, Fondazione IRCCS Policlinico San Matteo, Pavia, Italy; ^2^Biometric and Medical Statistics Unit, Fondazione IRCCS Policlinico San Matteo, Pavia, Italy; ^3^Pediatric Research Center Romeo ed Enrica Invernizzi, University of Milan, Milan, Italy; ^4^Department of Biomedical and Clinical Sciences “L. Sacco”, University of Milan, Milan, Italy; ^5^Risk Analysis and Genomic Epidemiology Unit, Istituto Zooprofilattico Sperimentale della Lombardia e dell’Emilia Romagna (IZSLER), Brescia, Italy; ^6^Department of Biosciences, University of Milan, Milan, Italy; ^7^Department of Biology and Biotechnology “L. Spallanzani”, University of Pavia, Pavia, Italy

**Keywords:** KPC, genomic epidemiology, MDR, *Klebsiella pneumoniae*, nosocomial outbreak, colistin resistance

## Abstract

The circulation of carbapenem-resistant *Klebsiella pneumoniae* (CRKP) is a significant problem worldwide. In this work we characterize the isolates and reconstruct the spread of a multi-clone epidemic event that occurred in an Intensive Care Unit in a hospital in Northern Italy. The event took place from August 2015 to May 2016 and involved 23 patients. Twelve of these patients were colonized by CRKP at the gastrointestinal level, while the other 11 were infected in various body districts. We retrospectively collected data on the inpatients and characterized a subset of the CRKP isolates using antibiotic resistance profiling and whole genome sequencing. A SNP-based phylogenetic approach was used to depict the evolutionary context of the obtained genomes, showing that 26 of the 32 isolates belong to three genome clusters, while the remaining six were classified as sporadic. The first genome cluster was composed of multi-resistant isolates of sequence type (ST) 512. Among those, two were resistant to colistin, one of which indicating the insurgence of resistance during an infection. One patient hospitalized in this period was colonized by two strains of CRKP, both carrying the *blaKPC* gene (variant KPC-3). The analysis of the genome contig containing the *bla*KPC locus indicates that the gene was not transmitted between the two isolates. The second infection cluster comprised four other genomes of ST512, while the third one (ST258) colonized 12 patients, causing five clinical infections and resulting in seven deaths. This cluster presented the highest level of antibiotic resistance, including colistin resistance in all 17 analyzed isolates. The three outbreaking clones did not present more virulence genes than the sporadic isolates and had different patterns of antibiotic resistance, however, were clearly distinct from the sporadic ones in terms of infection status, being the only ones causing overt infections.

## Introduction

*Klebsiella pneumoniae* (*Kp*) is ubiquitous in the environment, part of the normal intestinal microbiota in humans and capable of colonizing the skin and nasopharynx of healthy individuals (Podschun and Ullmann, 1998; Broberg et al., 2014). *Kp* can persist on abiotic surfaces of different origin through the synthesis of biofilm, which can also make bacteria resistant to the action of antimicrobial agents (Di Martino et al., 2003). In immunocompromised or debilitated hospitalized patients with severe underlying diseases, *Kp* causes urinary tract, respiratory tract and bloodstream infections (Podschun and Ullmann, 1998) as well as other less frequent diseases, including osteomyelitis, arthritis (Ghorashi et al., 2011), and meningitis (Ko et al., 2002; Tumbarello et al., 2006; Nordmann et al., 2009). *Kp* is responsible for roughly 12% of Gram-negative infections in hospital intensive care units (ICUs) in Europe (European Centre for Disease Prevention and Control [ECDC], 2016). *Kp* invasive infections are associated with high rates of morbidity and mortality due to the high prevalence of resistance to most available antimicrobial agents (Patel et al., 2008; Borer et al., 2009). This is an emerging concern in clinical care resulting in an increase of mortality rates and costs.

The most commonly used class of antibiotics against nosocomial infections is β-lactams, which includes penicillin derivatives, cephalosporins, monobactams and the most recently developed carbapenems. Frequent use and abuse of these drugs, combined with the transmissibility of resistance determinants mediated by mobile elements (plasmids, transposons, and other integrative conjugative elements), has contributed to the spread of resistance to β-lactams by *Kp* (Mathers et al., 2015; Navon-Venezia et al., 2017). In the last 20 years, the emergence of isolates resistant to carbapenems has limited the efficacy of this last line treatment option hampering the use of this whole class of antibiotics, with few alternatives (Mathers et al., 2015; Navon-Venezia et al., 2017). One of the most common mechanism of resistance to carbapenems in *Kp* is the *K. pneumoniae* carbapenemase (KPC). This is an Ambler molecular class A serine enzyme that is able to hydrolyze a broad variety of β-lactams. KPC carbapenemases are plasmid-encoded, they have been originally associated with the *Kp* clonal group 258 (CG258) (Samuelsen et al., 2009; Breurec et al., 2013) but are not limited to it, as a number of occurrences of strains of other sequence types (ST) carrying the gene have been reported (Giakkoupi et al., 2010; Qi et al., 2010; Tzouvelekis et al., 2013; Markovska et al., 2015; Oteo et al., 2016; Villa et al., 2016; Wei et al., 2016; Aires et al., 2017). KPC-carrying *Kp* strains have recently spread worldwide, with some countries, including Italy, being heavily affected. In Italy, in 2017, 33.9% of the *Kp* nosocomial infections were caused by KPC strains (Sabbatucci et al., 2017). Most of these strains belong to the CG258, which in Italy has been shown to have been imported on four occasions, giving rise to four Italian-subclades (Gaiarsa et al., 2015). The spread of carbapenem-resistant strains has led to the resurgence of the use of colistin, previously abandoned due to its nephrotoxicity and neurotoxicity (Javan et al., 2015; Velkov et al., 2018). This has however resulted in the emergence of colistin-resistant strains, both due to the insurgence of chromosomal mutations (Cannatelli et al., 2014; Olaitan et al., 2014) and to the acquisition of plasmid-encoded resistance genes (Di Pilato et al., 2016; Liu et al., 2016).

Whole genome sequencing (WGS) of bacterial isolates is increasingly used for epidemiological investigations (Sabat et al., 2013). The use of genomics in clinical settings as a routine tool could in the future be an important aid to the microbiologist to accurately identify and characterize outbreaks at early stages, and to identify transmission routes. However, only from the combination of typing data with clinical and demographic data the correct interpretation of the origin and evolution of an outbreak can be obtained (Van Belkum et al., 2007).

The present retrospective study analyzes a nosocomial outbreak, lasting 10 months (August 2015 – May 2016), caused by *K. pneumoniae* KPC, in an ICU and the period immediately before and after, for a total of 12 months. Thanks to the combination of genomic, microbiological and clinical data it was possible to reconstruct the epidemic event and to characterize the multiple unrelated strains that spread in mostly temporally non-overlapping periods.

## The Event

At the Fondazione IRCCS Policlinico San Matteo in Pavia, a 900-bed hospital in Northern Italy, cardio-respiratory patients are admitted to a specific Cardiorespiratory ICU where preoperative assessment, anesthetic treatment and intensive post-operative treatment of the patients undergoing cardiac and thoracic surgery are performed. This ICU has eight beds and is managed by 37 staff members. All ICU patients are subjected to surveillance through rectal swab at admission and once a week during the stay in the ward, to monitor for carbapenem-resistant *Enterobacteriaceae* colonization. In case of positivity, additional contact precautions are applied, which are interrupted only after three consecutive negative surveillance samples and are resumed in case of a single new positive screening swab.

**FIGURE 1:**
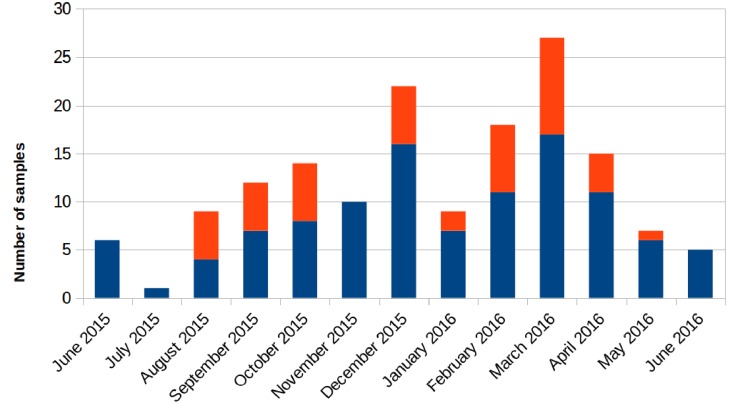
Number of samples positive for carbapenem-resistant *Klebsiella pneumoniae* from patients hospitalized in the ICU during the August 2015 – May 2016 period. Samples positive for rectal swab screening are colored in blue; samples showing positive infection are colored in orange.

Retrospectively, an increase in the number of carbapenem-resistant *Klebsiella pneumoniae* (CRKP) colonizations and infections in the ICU was observed in the period from August 2015 to May 2016. The first infection was reported on 15th August 2015. After this event, the number of infected and colonized patients gradually increased and peaked twice, in December 2015 and March 2016. When the first increase in cases was detected (December 2015), additional surveillance measures were undertaken: increase in the training of health personnel and in the use of disposable devices, more accurate daily cleaning, and increased passive surveillance. Environmental screenings were performed in the ward after the second peak in cases in March 2016, all resulting negative. No cohorting or spatial isolation of individual patients were applied. The situation returned to the norm, with no clinical infections, in June 2016 (Figure 1). The period from June 2015 to May 2016 was thus analyzed to understand the characteristics of the observed prolonged outbreak. A total of 426 patients were hospitalized in the ICU during that period and the procedure for rectal swab screening for Carbapenem-Resistant *Enterobacteriaceae* identified 23/426 CRKP-colonized patients (average age: 61.1 years; range: 27–80 years). Out of these 23 patients, 18 were negative at the admission time into the ICU. Two of the five patients (numbers 6 and 22) that were positive at admission at the ICU were previously hospitalized in other wards (respectively another ICU and Cardiac Surgery) were they became colonized. The remaining three patients (numbers 2, 4, and 7) were positive at the first swab, suggesting positivity before hospitalization. During the event 11/23 (47.8%) patients died, 2/23 (8.7%) were discharged for rehabilitation and 10/23 (43.5%) were transferred to other hospitalization facilities or hospital wards. Eleven of these 23 (47.8%) patients developed overt infections, with CRKP isolated from blood, respiratory tract, urine and wounds. Seven of the 11 infected patients died while at the ICU. The demographic and hospitalization characteristics of the 23 patients are reported in Table 1. During the same period, a total of eight other patients resulted infected by non-carbapenem resistant *Kp*, thus 58% of total *Kp* infections were CRKP.

**TABLE 1:** Table showing the hospitalization characteristics of all patients admitted to the ICU in the examined period that resulted positive to carbapenem-resistant *Klebsiella pneumoniae* at least once.

**Patient number**	**Admission date**	**Discharge date**	**Sample number**		**Outcome**
			**Surveillance**	**Clinical**	
1	May 24, 2015	June 17, 2015	3	0	Transfer to another hospital ward
2	June 17, 2015	June 23, 2015	2	0	Transfer to another hospital ward
3	July 31, 2015	September 16, 2015	13	1	Death
4	June 29, 2015	June 30, 2015	2	0	Transfer to another hospital ward
5	August 21, 2015	March 04, 2016	28	15	Death
6	July 11, 2015	September 18, 2015	1	1	Transfer to another hospital ward
7	October 08, 2015	October 23, 2015	3	1	Transfer to another hospital ward
8	October 14, 2015	November 17, 2015	6	2	Death
9	October 22, 2015	December 28, 2015	10	0	Death
10	October 31, 2015	December 17, 2015	7	0	Discharge for rehabilitation
11	November 24, 2015	December 03, 2015	2	0	Transfer to another hospital ward
12	November 26, 2015	December 31, 2015	9	4	Death
13	December 30, 2015	January 25, 2016	4	1	Transfer to another hospital ward
14	January 07, 2016	February 12, 2016	6	0	Death
15	January 08, 2016	May 06, 2016	17	2	Death
16	January 15, 2016	March 22, 2016	11	10	Death
17	January 22, 2016	March 21, 2016	8	0	Death
18	February 06, 2016	March 20, 2016	6	0	Death
19	February 26, 2016	April 03, 2016	6	8	Death
20	March 01, 2016	March 15,2016	3	0	Transfer to another hospital ward
21	March 31, 2016	May 25, 2016	5	1	Discharge for rehabilitation
22	April 04, 2016	April 08, 2016	1	0	Transfer to another hospital ward
23	April 11, 2016	April 26, 2016	3	0	Transfer to another hospital ward

## Materials and Methods

### Ethics Statement

The study was designed and conducted in accordance with the Helsinki declaration and approved by the Ethics Committee of Fondazione IRCCS Policlinico San Matteo in Pavia, Italy.

### Bacterial Strain Identification and Susceptibility Testing

During the entirety of the event, all surveillance rectal swabs were sent to the Microbiology and Virology Unit and were directly plated on chromID CARBA Agar (BioMérieux, Marcy-l’Étoile, France) to screen for the presence of carbapenemase-producing *Enterobacteriaceae*. In the same period, all clinical specimens (blood samples, bronchial aspirates, bronchoalveolar wash, urine samples, and wound swabs) were all cultured in parallel on all the following media: Columbia Blood Agar with 5% sheep blood, chocolate agar, selective media or on Schaedler agar and 5% sheep blood (BioMérieux SA, Marcy-l’Étoile, France) anaerobically and incubated at 37°C overnight. Colonies suspected to be *Kp* based on morphology were identified with the MALDI Biotyper 3.1 system based on Matrix-Assisted Laser Desorption Ionization time-of-flight (MALDI-TOF) (Bruker Daltonics, Bremen, Germany).

We selected a subset of the *Kp* isolates from surveillance and clinical samples based on sample type, collection date and resistance profile, to perform additional characterization. Antibiotic susceptibility testing and minimum inhibitory concentrations (MICs) determinations were performed using the BD Phoenix 100 automated system (Becton, Dickinson and Company, Franklin Lakes, NJ, United States) and interpreted following the clinical breakpoints of the version 6.0 of the European Committee on Antimicrobial Susceptibility Testing [EUCAST] (2016)^1^. Tigecycline and carbapenems MICs were confirmed by *E*-test strips (BioMérieux, Marcy-l’Étoile, France). As recommended by European Committee on Antimicrobial Susceptibility Testing [EUCAST] (2016)^2^ and the European Centre for Disease Prevention and Control [ECDC] (2016)^3^, colistin MICs were confirmed performing the broth microdiluition (MIC-Strip Colistin (MERLIN Diagnostika GmbH, Germany).

### DNA Extraction and Genome Sequencing

Total DNA extraction was performed for the selected isolates using the QIAamp DNA mini kit (Qiagen, Italy) according to manufacturer’s instructions. DNA was sequenced using the Illumina MiSeq platform (Illumina Inc., San Diego, CA, United States), with paired-end runs of 2 × 250 bp, after Nextera XT library preparation.

### Genomic Analyses

Sequencing reads were quality checked using FastQC^4^ and trimmed using the Trimmomatic software (Bolger et al., 2014). SPAdes-3.10.1 (Bankevich et al., 2012) was then used to assemble the pair-end reads using the accurate setting. Multilocus sequence typing (MLST) profiles and virulence/resistance gene variants were determined *in silico* using the Kleborate tool^5^. Presence of the *mcr* gene was tested using ResFinder Version 3.1.0 (database updated to February 20, 2019) (Zankari et al., 2012). Plasmid content was characterized using PlasmidFinder Version 2.0.1 (database updated to November 20, 2018) (Carattoli et al., 2014), while the contigs containing the *bla*KPC gene were compared using the BLASTn and the Mauve software (Darling et al., 2010) and subjected to Maximum Likelihood phylogeny using RAxML (Stamatakis, 2014) with the GAMMA substitution model, considering the Ascertainment bias and applying the Lewis correction (Lewis, 2001). The tree topology reliability was tested using 100 bootstrap replicates.

To estimate genomic variability, the obtained genome sequences were added to a selected dataset of *K. pneumoniae* genomes extracted from the PATRIC database (Wattam et al., 2016). PATRIC genomes were selected, using an in-house script, to be the closest in genomic distance to our strains. In detail, each genome was compared to all PATRIC database genomes using Mash (Ondov et al., 2016) and the 50 best hits were selected. All the obtained best hits lists were merged removing duplication, to obtain the final genomic dataset. CoreSNPs were extracted from the resulting dataset following a published method (Gaiarsa et al., 2015). Briefly, the Mauve software (Darling et al., 2010) was used to align all novel genomes and the similar PATRIC genomes to a well-characterized complete genome reference [NZ_CP006923 (DeLeo et al., 2014)]. Individual alignments were merged using a Python script to obtain a multi-alignment file, allowing to extract coreSNPs (defined as variations of a single nucleotide flanked on each side by two nucleotides conserved in all the genomes analyzed).

**FIGURE 2:**
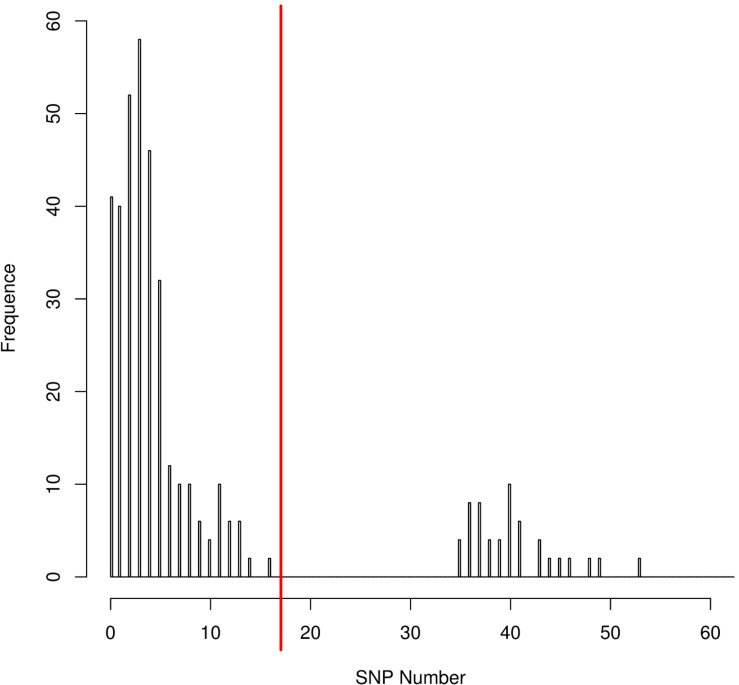
CoreSNPs distribution between genome pairs. Pairs of genomes within a distance of 16 SNPs (SNPs threshold) were considered as part of the same transmission cluster.

The distribution of the coreSNPs distances among strains was visualized using the software R Version 3.2.3 (R Core Team, 2013) and the coreSNP cut-off threshold was determined manually by looking at the graph and finding that there were two groups of genome pairs, those with less than 15 SNPs and those with more than 35 SNPs (Figure 2). The distribution allowed us to safely infer that two genomes could be considered as part of the same transmission cluster if their distance in number of coreSNPs was lower than the 15 SNPs threshold value. The coreSNPs alignment was used to perform a phylogenetic analysis using the software RAxML (Stamatakis, 2014) with the GAMMA substitution model, considering the Ascertainment bias and applying the Lewis correction (Lewis, 2001). The tree topology reliability was tested using 100 bootstrap replicates.

The genomes of the epidemic clusters were investigated also in order to detect possible unique genomic characteristics. A SNP based phylogeny was generated for each cluster, using the same approach as the global phylogeny, and adding to each cluster the genome not part of the cluster that resulted the closest in the global phylogeny. The phylogenies and the genomic alignments of each cluster and their outgroup were used as inputs for a recombination analysis with the software ClonalFrameML (Didelot and Wilson, 2015). Furthermore, the presence and abundance of Insertion Sequences (IS) was tested using the software ISSeeker (Adams et al., 2016) against all IS retrieved from the Issaga database (Varani et al., 2011) using “*Klebsiella*” as organism keyword (on date July 17, 2019).

## Results

An outbreak of CRKP occurred in the cardio-respiratory ICU from August 2015 to May 2016, involving 23 patients (12 colonized, 11 infected). In order to characterize the event, we retrieved all the CRKP strains isolated during the event and in the 2 months before. We obtained a total of 144 samples, 98/144 (68.1%) surveillance samples and 46/144 (31.9%) clinical samples (16 blood cultures, 12 bronchial aspirates, 1 bronchoalveolar wash, 8 urines, and 9 wound swabs). A total of 32/144 *K. pneumoniae* isolates, from 22 rectal swabs and 10 clinical specimens, were selected based on sample types and collection date, including at least one per each of the 23 positive patients, for antibiotic resistance characterization and genome sequencing (Table 2). The results of the antibiotic susceptibility tests are reported in Supplementary Table S1 and show that all the isolates are resistant to at least one carbapenem: 32/32 to Ertapenem, 31/32 to Meropenem, 29/32 to Imipenem. Moreover, 31 isolates are resistant to aminoglycosides (25 to Amikacin, 22 to Gentamicin, 28 to Tobramycin). Twenty isolates are resistant to Fosfomycin (MICs > 64 mg/L), 20 to Colistin (MICs ranging from 4 to >64 mg/L) and three are resistant to Tigecycline (MICs > 2 mg/L). Only one strain from a surveillance sample (genome ID: 1880), resulted to be resistant to all five classes of antibiotics.

**TABLE 2:** Description of the 32 *Klebsiella pneumoniae* KPC isolates selected for the genomic characterization.

**Genome ID**	**Patient number**	**Sample date**	**Sample type**	**Sequence type**	**KPC-type**
1753	1	June 02, 2015	rectal swab	ST512	KPC-3
1758	2	June 23, 2015	rectal swab	ST258	KPC-2
1760	4	June 29, 2015	rectal swab	ST258	KPC-2
1826	3	August 11, 2015	rectal swab	ST45	KPC-2
1845	5	September 01, 2015	rectal swab	ST512	KPC-3
1870	5	September 14, 2015	wound swab	ST512	KPC-3
1873	5	August 31, 2015	blood	ST512	KPC-3
1880	6	September 15, 2015	rectal swab	ST512	KPC-3
1897	7	October 08, 2015	rectal swab	ST512	KPC-3
1935	8	October 26, 2015	bronchial aspirate	ST512	KPC-3
1955	9	November 10, 2015	rectal swab	ST512	KPC-3
1961	10	November 17, 2015	rectal swab	ST512	KPC-3
1987	11	December 01, 2015	rectal swab	ST258	KPC-3
1998	5	December 01, 2015	rectal swab	ST3985	KPC-3
2003	12	December 08, 2015	rectal swab	ST258	KPC-3
2018	12	December 29, 2015	bronchial aspirate	ST258	KPC-3
2066	13	January 15, 2016	bronchial aspirate	ST258	KPC-3
2079	16	January 26, 2016	rectal swab	ST258	KPC-3
2106	5	February 02, 2016	rectal swab	ST512	KPC-3
2110	14	February 02, 2016	rectal swab	ST258	KPC-3
2133	17	February 16, 2016	rectal swab	ST258	KPC-3
2137	16	February 07, 2016	blood	ST258	KPC-3
2165	19	March 01, 2016	bronchial aspirate	ST258	KPC-3
2174	19	March 14, 2016	blood	ST258	KPC-3
2176	20	March 08, 2016	rectal swab	ST258	KPC-3
2182	18	March 15, 2016	rectal swab	ST258	KPC-3
2183	15	March 15, 2016	rectal swab	ST258	KPC-3
2186	16	March 19, 2016	wound swab	ST258	KPC-3
2205	22	April 04, 2016	rectal swab	ST512	KPC-3
2218	23	April 19, 2016	rectal swab	ST258	KPC-3
2221	21	April 19, 2016	rectal swab	ST258	KPC-3
2228	21	April 24, 2016	blood	ST258	KPC-3

Whole-genome sequences were obtained for the 32 selected strains using Illumina technology and assembled into draft genomes after read polishing. Genomes resulted to be of good quality (average N50: 191,110; average number for contigs above 500 bp = 116; average genome size (5,671,622 bp, see Supplementary Table S2 for complete genome characteristics). Genomes have been submitted to EMBL and are accessible under accession PRJEB32609 (ERP115310). Genomes were characterized by calculating the MLST, detecting antibiotic resistance determinants and virulence factors (see Supplementary Tables S3, S4 for complete results). The most prevalent ST was ST258 (*n* = 19, 59.4%), followed by ST512 (*n* = 11, 34.4%). One isolate was found to belong to ST45 (3.1%). One new ST was found, a single-locus variant (-SLV) of ST940 (genome 1998 from patient 5, now registered as ST3985). Among the 5 patients with more than one isolate sequenced, one resulted to be colonized by isolates of different STs (patients 5).

Results of the antibiotic resistance factors analysis (Supplementary Table S3) were grouped in 12 drug classes according to the ARG-ANNOT database (Gupta et al., 2014), with β-lactamases divided into the six Lahey classes (Bush and Jacoby, 2010). All 32 analyzed isolates carried at least one ESBL gene (3.1% CTX-M-15, 3.1% SHV-1, 87.5% SHV-11, 6.3% SHV-12) and/or inhibitor-resistant β-lactamase genes (87.5% TEM-54 and 3.1% TEM-122). All the isolates were confirmed to be resistant to carbapenems (9.4% KPC-2 and 90.6% KPC-3). Kleborate identified colistin resistance as truncation or loss of *mgrB* gene in 20 isolates (62.5%), if less than 90% of the reference gene was covered by sequencing reads. Manual analysis allowed to detect the presence of an Insertion Sequence (IS5-like) interrupting *mgrB* in two resistant isolates (1880 and 1753). The interruption due to this mobile element has been previously reported (Cannatelli et al., 2014). All the other resistant isolates, belonging to cluster 3, exhibited a frameshift mutation in *mgrB* due to a 10 nucleotide insertion in position 109 of the gene. Presence of *mcr* genes was investigated with ResFinder, but they were never detected. Colistin antibiogram and genomic data resulted to be coherent for all clones, with two exceptions, clone 1760 indicated as resistant in *Kleborate* but resulting sensitive at the microdilution test and clone 1870 resistant to the phenotypic test but not to the genomic analysis.

Results of the virulence determinants presence show that only 4/32 strains (12.5%) contained the *ybt* locus, which encodes the biosynthesis pathway of the siderophore yersiniabactin (Lam et al., 2018). None of the strains were found to harbor other siderophores (aerobactin and salmochelin were screened). All the four *ybt* positive strains were isolated from surveillance rectal swabs of different patients and were sporadic cases. Two of them (genomes 1758 and 1760) showed *ybt* allele 13, carried by integrative conjugative element: ICEKp 2. The other two isolates (genomes 1826 and 1998) showed respectively the variants: *ybt* 10 (carried by ICEKp 4) and *ybt* 4 (plasmid-borne allele). The association between the mobile genetic elements found and the allelic variants of *ybt* gene is in agreement with previous observations (Lam et al., 2018). The biosynthetic pathway for the production of the toxin colibactin, as well as the virulence factor *rmp*A/*rmp*A2 (responsible for the expression of the hypermucose-viscous phenotype) were absent from all 32 strains. Finally, using the Kleborate tool we identified three distinct K-loci. The most common K-locus was KL107 (*n* = 28, 90.3%), followed by KL106 (*n* = 2, 6.5%) and KL24 (*n* = 1, 3.2%). As expected from the literature (Shu et al., 2009), they were found to be associated with *wzi* alleles 154, 29, and 101, respectively. K-locus was not typable for one isolate (1998) (see Supplementary Table S4 for full results).

**FIGURE 3:**
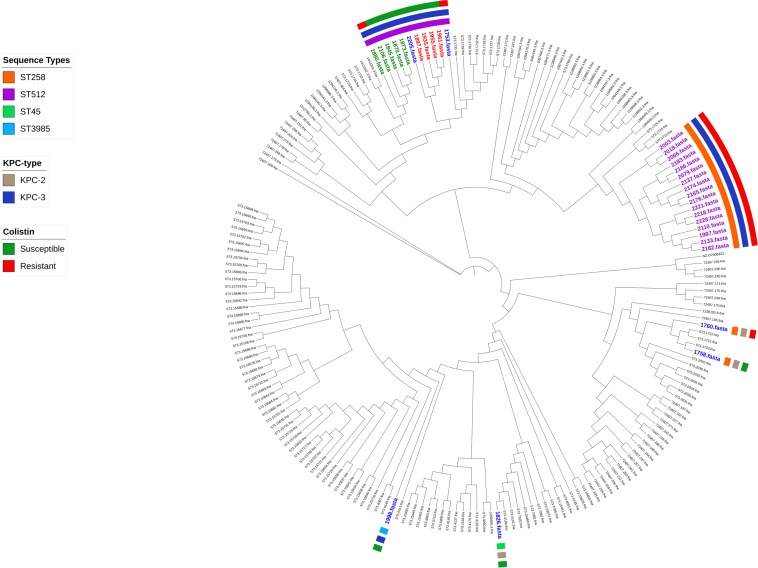
Phylogenetic tree based on coreSNPs from 32 *Klebsiella pneumoniae* KPC strains isolated from the Intensive Care Unit and 172 related genomes retrieved from the PATRIC database. Sporadic strains are highlighted in blue; in green, red, and violet are the genomes belonging to three monophyletic clusters (distance in SNPs < 16).

**FIGURE 4:**
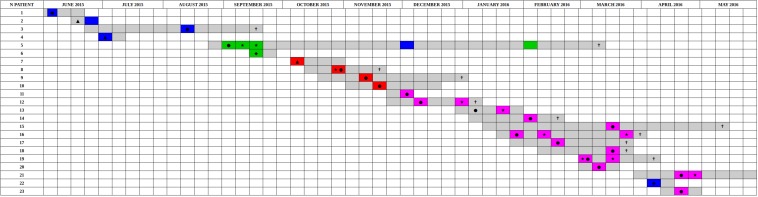
Timeline of the presence of carbapenem-resistant *Klebsiella pneumoniae* positive patients in the ICU in the period from June 2015 to May 2016. The periods of hospitalization are colored in gray. The other colors identify the sequenced samples based on genomic characterization. The colors green, red, and violet correspond to the three clusters identified based on SNPs distance, are reported sporadic cases in blue. Samples obtained from clinical infections (not surveillance swabs) are indicated by stars. The crosses indicate patients death. Black circles indicate the first positive CRKP rectal swab for each patient, negative at the admission. Black triangles indicate the first positive CRKP rectal swab for patients already CRKP-colonized at the admission time into the hospital. Black diamonds indicated the patients that became positive in other hospital wards.

The distribution of the coreSNPs distances among the 32 sequenced genomes was calculated and plotted to determine a threshold cut-off indicating epidemiological relatedness. A clear threshold was detected at 16 coreSNPs (Figure 2), allowing to determine the presence of three clades of genomes. Within each clade, all genomes have reciprocal coreSNP distances lower than the calculated threshold. A global coreSNPs maximum-likelihood phylogeny was performed, including the 32 genomes investigated in this work and 172 related genomes extracted from the PATRIC database, in order to contextualize our strains within the surrounding *Kp* diversity. The genomes with coreSNPs distance below the threshold clustered in three monophyletic clades (Figure 3 for the cladogram, see also Supplementary Figure S1 that shows subtrees of the three clusters) and were thus considered part of three separate outbreak clusters (green, red, and violet clusters in Figures 3, 4). Five of the other six genomes do not cluster with other genomes of the outbreak and are thus considered sporadic cases. The last remaining genome (2205) is the phylogenetic sister group of cluster 2, but presents a relatively high number of coreSNPs with the other genomes of the cluster (average 42 coreSNPs), and was thus considered as a separate, sporadic, case.

Comparative genomic analyses were performed within the three clades and to compare them to their nearest neighbor, showing very limited genomic variation. No recombination was detected at the origin of any of the three epidemic clades (Supplementary Figure S2). IS content of isolates of each of the three outbreaking clusters was compared using ISfinder. The IS content resulted to be stable within each cluster, while each cluster appears to have more ISs than the evolutionary closest sporadic isolates. Specifically, isolates of Cluster 1 have IS of families ISL3 and IS1 that are absent in the sporadic sister group. Isolates of cluster 2 present IS66 sequences, absent in the evolutionary closest sporadic isolate. Isolates of cluster 2 are also richer in ISL3 and IS6 sequences than their neighbor and those of cluster 3 are richer in IS5 and IS6. It must be noted however that the accuracy, especially quantitatively, of such an analysis on draft genomes is limited, due to the difficulties in assemblying IS sequences (Supplementary Figure S3).

## Discussion

Here we present the results of the integrated characterization of the CRKP strains isolated during a 1-year period (10 months outbreak plus 2 months before) in the cardio-respiratory ICU of the San Matteo hospital in Pavia, Northern Italy. Retrospectively, starting in August 2015, we observed a strong increase in CRKP isolated from routine surveillance rectal swabs, taken from each ICU patient every week indicating a high number of colonized patients. We also observed an increase in CRKP isolations from clinical samples, indicating overt infections. This trend continued until June 2016, with a total of 23 colonized patients, among which 11 infected, prompting the investigation presented here. Antibiotic resistance characterization coupled with genome sequencing of 32 isolates from these 23 patients (Table 2), collected in the outbreak period and in the 2 months before, allowed the characterization of the extended outbreak. Most strains were found to belong to CG258, one of the most widespread KPC-bearing group in Italy and worldwide (Gaiarsa et al., 2015; Onori et al., 2015).

Performing a SNPs distance analysis (Figure 1) we found that twenty-six of the 32 isolates resulted to belong to three genome clusters (coreSNPs < 16) that appeared in the ICU, infected multiple patients, and then disappeared, with one single strain lasting for most of the event (Figure 4, see also Supplementary Figure S1 for phylogenetic reconstructions of the three clusters). The remaining six genomes resulted to be unrelated and were consequently classified as sporadic isolates, as they all showed more than 35 coreSNPs with all the other analyzed genomes. This result is coherent with a recent multicenter study that proposed to set a threshold for SNPs distance in *Kp* outbreak at 21 (David et al., 2019). Interestingly, four of the six sporadic isolates represent the strains characterized in the 2 months before the outbreak (June–August 2015, see Figure 4), indicating that multiple strains were present in the ICU, but they were only responsible for colonization, did not cause clinical infections, nor they spread to multiple patients. The six sporadic isolates are characterized by variable levels of antibiotic resistance (number of resistance classes determined with Kleborate ranging from 3 to 11). Two of them are resistant to colistin (genomes ID: 1753, 1760). In terms of virulence, the only isolates that present yersiniabactin are four of the six sporadic isolates, all isolated from colonized, not infected, patients. Most sporadic isolates belong to the CG258 (*n* = 4), one belongs to ST45, already reported as a KPC-bearing strain in Italy (Cristina et al., 2016), and the final one to a novel ST, variant of ST940, now classified as ST3985. These sporadic isolates could be epidemiologically important, acting as a “reservoir” for the spread of the mobile element carrying *bla*KPC.

The strain responsible for the first cluster of isolations, belonging to ST512, was isolated from patient 5 from a screening sample in August 2015 and then from clinical samples in September. The patient remained colonized until his death in March. The isolates of this cluster are characterized by the resistance to 10 antibiotic classes, remaining susceptible to colistin, with two exceptions. The first one (genome ID: 1880) is the only strain of the cluster, belonging to patient 6, which present a correspondence between phenotypic (MIC > 64 mg/L) and genotypic (loss/truncation of *mgr*B gene) colistin resistance. The other colistin-resistant strain of the cluster (genome ID: 1870) was the third of four in the temporal series isolated from patient 5 and resulted phenotypically colistin-resistant (MIC = 4 mg/L), even though no colistin-resistance mutation/gene was detected in the genome. In order to investigate the presence of other mutations potentially causing colistin resistance, the genomes of the isolates belonging to patient 5 were manually examined, detecting 2 SNPs unique to the colistin-resistant isolate. These SNPs are co-localized in an intergenic region upstream of the *pca* operon, potentially responsible for the β-ketoadipate pathway. Understanding whether these SNPs could be related to colistin resistance would require additional investigations.

One additional CRKP was isolated from patient 5 on December 1st. This isolate was unrelated to those of cluster 1, belonging to ST3985 (a novel SLV of ST940). In order to evaluate whether this strain acquired the KPC plasmid from the ST512 strain co-present in patient 5, we compared the contigs harboring the KPC gene, performing a phylogenetic analysis including contigs from isolates of cluster 1, from the ST3985, and from another sporadic isolate (1753), as control. We found that the KPC-harboring contig from the ST3985 was divergent from all identical contigs retrieved from the ST512 isolates belonging to cluster 1, but also from the sporadic control, which resulted sister group of the isolates of the cluster (Supplementary Figure S4).

Four isolates were grouped in cluster 2, belonging again to ST512. This clone was short-lived but was transmitted from patient 7 to three other patients in the course of 6 weeks and caused one clinical infection. The isolates of this cluster are resistant to most antibiotics, retaining susceptibility to colistin and tigecycline. Two of four isolates resulted phenotypically sensitive to aminoglycosides, while genome analysis are only in partial agreement, indicating that all four strains carry the *aph3-Ia* gene, which should confer resistance to this class of antibiotics.

The largest of the clusters is represented by 17 strains, isolated from 12 patients during 5 months, causing five clinical infections and resulting in three deaths. The isolates of this cluster are those characterized by the highest level of resistance, retaining susceptibility only to tigecycline. Genomic analysis agrees with this result indicating the presence of resistance genes against 11 antibiotic classes, one more than the two other clusters. Indeed, all the isolates of this cluster resulted resistant to colistin with phenotypic tests, and the analysis of the genome content using Kleborate highlighted a possible resistance-causing truncation in the *mgrB* gene in all 17 strains. Manual analysis allowed to detect a frameshift mutation in *mgrB* due to a ten nucleotide insertion in position 109 of the gene.

## Conclusion

Over the course of 1 year, nine different strains of Carbapenem resistant *K. pneumoniae* were isolated in the ICU from 23 patients, 12 only colonized, 11 infected. Three strains colonized multiple patients, were the cause of all the seven clinical infections reported in the ICU in this period and were responsible for the outbreak that lasted for 10 months. The three outbreaking strains did not present more virulence genes than the six sporadic isolates, four of which were actually the only ones exhibiting yersiniabactin. In terms of antibiotic resistance, both outbreaking and sporadic strains were phenotypically resistant to most classes, a result confirmed by strong repertoires of resistance genes. A clear correlation between antibiotic resistance profiles and number of colonizations or infections is thus not clearly evident. Our results highlight the importance of the overall environmental context, possibly more than the intrinsic characteristics of a strain, in determining the spread of different CRKP isolates.

## Data Availability Statement

The genomes generated for this study can be found in the EMBL EBI repository, under the PRJEB32609 entry.

## Ethics Statement

Neither ethics committee approval, nor informed consent were required as all collected data were fully anonymized, there was no contact with patients and/or their families and no interventions or changes to treatment and management were made, in accordance with institutional guidelines.

## Author Contributions

CF, CB, PM, and DS designed the project. MC and PC performed the microbiological analyses. ES performed the genome sequencing. CF, SG, and FC performed the bioinformatic analyses. CF, MC, and DS performed the epidemiological analyses. CF, MC, SG, and DS drafted the manuscript. CB, PM, and DS finalized the manuscript. All authors read and approved the final manuscript.

## Conflict of Interest

The authors declare that the research was conducted in the absence of any commercial or financial relationships that could be construed as a potential conflict of interest.

## Footnotes

1 http://www.eucast.org/fileadmin/src/media/PDFs/EUCAST_files/Breakpoint_tables/v_6.0_Breakpoint_table.xls

2 http://www.eucast.org/fileadmin/src/media/PDFs/EUCAST_files/General_documents/Recommendations_for_MIC_determination_of_colistin_March_2016.pdf

3 https://ecdc.europa.eu/sites/portal/files/media/en/publications/Publications/enterobacteriaceae-risk-assessment-diseases-caused-by-antimicrobial-resistant-microorganisms-europe-june-2016.pdf

4 www.bioinformatics.babraham.ac.uk/projects/fastqc/

5 https://github.com/katholt/Kleborate

## Supplementary Material

FIGURE S1 Recombination analysis of Cluster 1 **(A)**, Cluster 2 **(B)** and Cluster 3 **(C)**. The analysis was performed using the software ClonalFrameML and including the evolutionary closest sporadic genome as outgroup.

FIGURE S2 Insertion sequences (IS) content of the three epidemic clusters. The evolutionary closest sporadic genome was added as a reference, last in order in the figure for each cluster.

FIGURE S3 Phylogenetic trees of the three genomic clusters [Cluster 1 in panel **(A)**, Cluster 2 in panel **(B)** and Cluster 3 in panel **(C)**], based on coreSNPs from the global alignment described in Figure 1, determined using RAxML, with 100 bootstrap, showing branch lengths and patients numbers. For each cluster, the sister group genome, as determined by the tree in Figure 1 was used as outgroup.

FIGURE S4 Phylogenetic comparison of the KPC-bearing contigs of the 32 isolates. The contig from isolate 1998, the ST3985 genome, is clearly distinct from the contigs from the ST512 strains belonging to Cluster 1 (1897, 1935, 1955, 1961).

TABLE S1 Antimicrobial susceptibility profiles determined using the BD Phoenix 100 System on the 32 investigated *Klebsiella pneumoniae* strains.

TABLE S2 Characteristics of the sequenced genomes.

TABLE S3 Predicted antimicrobial susceptibility profiles determined using the Kleborate tool on 32 *Klebsiella pneumoniae* KPC isolates.

TABLE S4 Factors of the 32 *Klebsiella pneumoniae* KPC isolates.
