# Supplementary figures and images for: Multiple Klebsiella pneumoniae KPC Clones Contribute to an Extended Hospital Outbreak
Source: Front Microbiol. 2019 Nov 29;10:2767. doi: 10.3389/fmicb.2019.02767 (PMC6896718; doi:10.3389/fmicb.2019.02767)

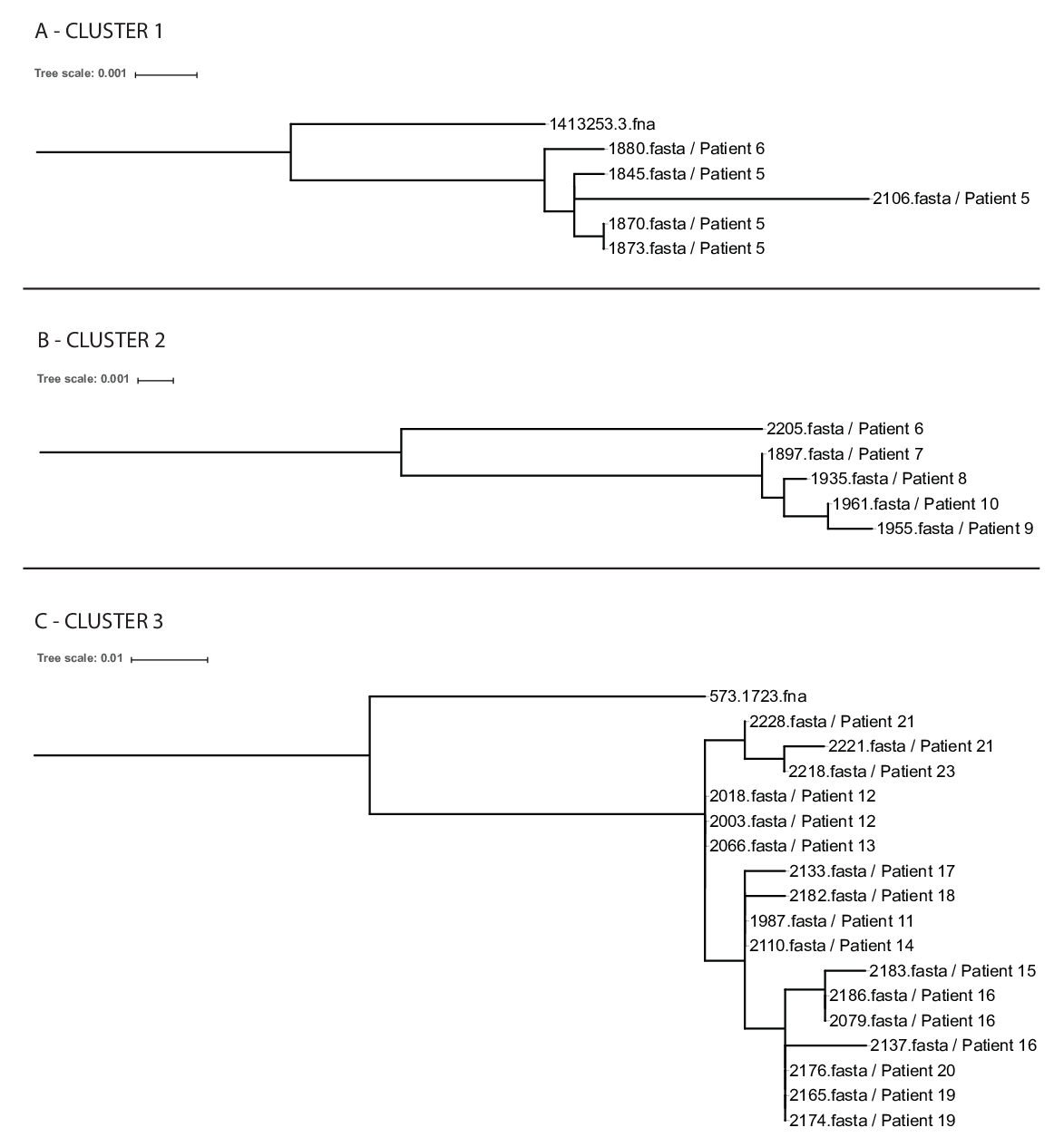

Supplement: FIGURE S1 — Recombination analysis of Cluster 1 (A), Cluster 2 (B) and Cluster 3 (C). The analysis was performed using the software ClonalFrameML and including the evolutionary closest sporadic genome as outgroup. [file Image_1.TIFF]

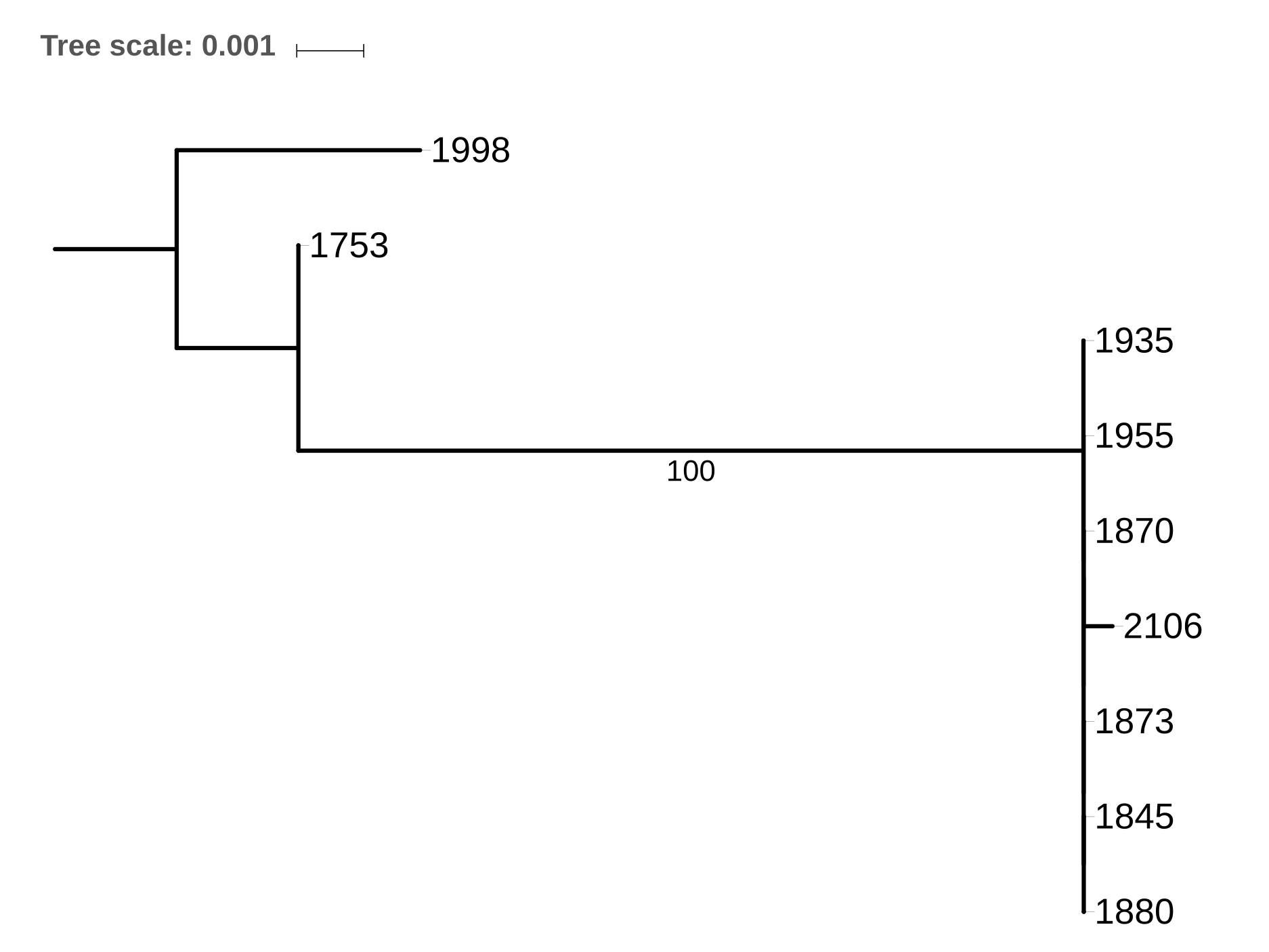

Supplement: FIGURE S2 — Insertion sequences (IS) content of the three epidemic clusters. The evolutionary closest sporadic genome was added as a reference, last in order in the figure for each cluster. [file Image_2.TIFF]

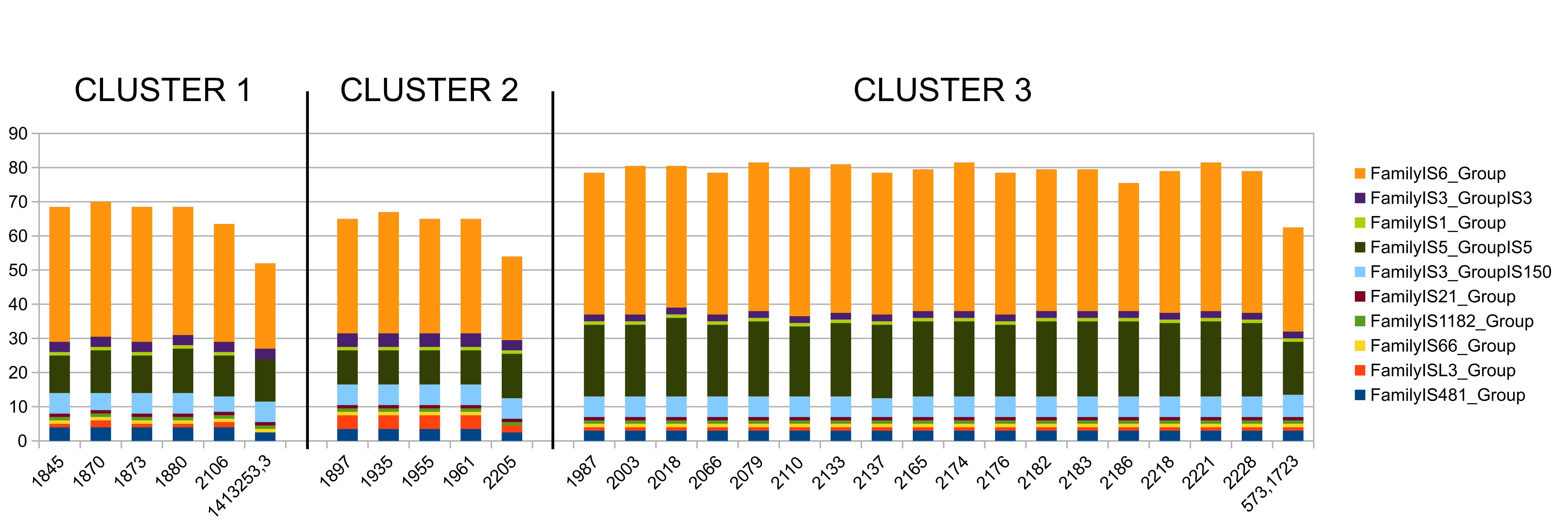

Supplement: FIGURE S4 — Phylogenetic comparison of the KPC-bearing contigs of the 32 isolates. The contig from isolate 1998, the ST3985 genome, is clearly distinct from the contigs from the ST512 strains belonging to Cluster 1 (1897, 1935, 1955, 1961). [file Image_4.TIFF]
