# Supplementary material for: Multiple Klebsiella pneumoniae KPC Clones Contribute to an Extended Hospital Outbreak
Source: Front Microbiol. 2019 Nov 29;10:2767. doi: 10.3389/fmicb.2019.02767 (PMC6896718; doi:10.3389/fmicb.2019.02767)
Supplement: TABLE S1 — Antimicrobial susceptibility profiles determined using the BD Phoenix 100 System on the 32 investigated Klebsiella pneumoniae strains. [file Table_1.DOCX]

Supplementary Table 1. Antimicrobial susceptibility profiles determined using the BD Phoenix 100 System on the 32 investigated *Klebsiella pneumoniae* strains. S, Susceptible; I, Intermediate; R, Resistant; ND, Not Determined. MIC breakpoints (mg/L): Amikacin: S≤8, R>16; Gentamicin: S≤2, R>4; Tobramycin: S≤2, R>4; Amoxicillin clavulanate: S≤8, R>8; Ampicillin: S≤8, R>8; Piperacillin: S≤8, R>8; Aztreonam: S≤1, R>4; Cefuroxime: S≤8, R>8; Cefotaxime: S≤1, R>2; Ceftazidime: S≤1, R>4; Cefepime: S≤1, R>4; Ertapenem: S≤0.5, R>0.5; Imipenem: S≤2, R>4; Meropenem: S≤2, R>8; Piperacillin/Tazobactam: S≤8, R>16; Ciprofloxacin: S≤0.25, R>0.5; Levofloxacin: S≤0.5, R>1; Colistin: S≤2, R>2; Tigecycline: S≤1, R>2; Fosfomycin: S≤32, R>32; Trimethoprim/Sulfamethoxazole: S≤2, R>4.

| **Genome ID** | **Patient number** | **ST** | **Cluster** | **Amikacin** | **Gentamicin** | **Tobramycin** | **Amoxicillin clavulanate** | **Ampicillin** | **Piperacillin** | **Aztreonam** | **Cefuroxime** | **Cefotaxime** | **Ceftazidime** | **Cefepime** | **Ertapenem** | **Imipenem** | **Meropenem** | **Piperacillin/**  **Tazobactam** | **Ciprofloxacin** | **Levofloxacin** | **Colistin** | **Tigecycline** | **Fosfomycin** | **Trimethoprim/**  **Sulfamethoxazole** |
| --- | --- | --- | --- | --- | --- | --- | --- | --- | --- | --- | --- | --- | --- | --- | --- | --- | --- | --- | --- | --- | --- | --- | --- | --- |
| 1753 | 1 | ST512 | Sporadic | R, >16 | S, 2 | R, >4 | R, >8/2 | R, >8 | R, >16 | R, >16 | R, >8 | R, >4 | R, >8 | R, ND | R, >1 | R, >32 | R, >32 | R, >16/4 | R, >1 | R, >2 | R, 16 | S, 0.75 | R, 64 | S, <=1/19 |
| 1758 | 2 | ST258 | Sporadic | R, >16 | R, 32 | R, >4 | R, >8/2 | R, >8 | R, >16 | R, >16 | R, >8 | R, >4 | R, >8 | R, >8 | R, >1 | ND | R, >32 | R, >16/4 | R, >1 | R, >2 | S, 1 | ND | R, >64 | S, <=1/19 |
| 1760 | 4 | ST258 | Sporadic | R, >16 | I, 4 | R, >4 | R, >8/2 | R, >8 | R, >16 | R, >16 | R, >8 | R, >4 | R, >8 | R, >8 | R, >1 | R, >8 | R, >8 | R, >16/4 | R, >1 | R, >2 | S, 1 | S, 0.512 | S, <=16 | R, >4/76 |
| 1826 | 3 | ST45 | Sporadic | R, >16 | R, >4 | R, >4 | R, >32/2 | R, >8 | R, >16 | ND | R, >8 | R, >4 | R, >8 | R, >8 | R, >1 | I, 8 | R, >8 | R, >16/4 | R, >1 | R, >2 | S, 0.5 | ND | R, >64 | S, <=1/19 |
| 1845 | 5 | ST512 | Green | I, 16 | S, 2 | R, >4 | R, >32/2 | R, >8 | R, >16 | ND | R, >8 | R, >4 | R, >8 | R, >8 | R, >1 | R, >8 | R, >8 | R, >16/4 | R, >1 | R, >2 | S, 1 | R, >2 | R, >64 | R, >4/76 |
| 1870 | 5 | ST512 | Green | R, >16 | R, >4 | R, >4 | R, >32/2 | R, >8 | R, >16 | ND | R, >8 | R, >4 | R, >8 | R, >8 | R, >1 | R, >8 | R, >8 | R, >16/4 | R, >1 | R, >2 | R, 4 | I, 2 | S, <=16 | R, >4/76 |
| 1873 | 5 | ST512 | Green | I, 16 | S, <=1 | R, >4 | R, >32/2 | R, >8 | R, >16 | ND | R, >8 | R, >4 | R, >8 | R, >8 | R, >1 | R, >8 | R, >8 | R, >16/4 | R, >1 | R, >2 | S, 0.5 | R, >2 | S, 32 | R, >4/76 |
| 1880 | 6 | ST512 | Green | I, 16 | S, 2 | R, >4 | R, >32/2 | R, >8 | R, >16 | ND | R, >8 | R, >4 | R, >8 | R, >8 | R, >1 | R, >8 | R, >8 | R, >16/4 | R, >1 | R, >2 | R, 64 | R, >2 | R, 64 | R, >4/76 |
| 1897 | 7 | ST512 | Red | S, 8 | R, ND | S, 2 | R, >32/2 | R, >8 | R, >16 | ND | R, >8 | R, >4 | R, >8 | R, >8 | R, >1 | R, >32 | R, >32 | R, >16/4 | R, >1 | R, >2 | S, <=1 | S, ND | R, >64 | R, >4/76 |
| 1935 | 8 | ST512 | Red | S, 8 | R, ND | S, 2 | R, >32/2 | R, >8 | R, >16 | ND | R, >8 | R, >4 | R, >8 | R, >8 | R, >1 | R, >32 | R, >32 | R, >16/4 | R, >1 | R, >2 | S, 1 | S, 0.50 | R, >64 | R, >4/76 |
| 1955 | 9 | ST512 | Red | S, <=4 | S, 2 | S, 2 | R, >32/2 | R, >8 | R, >16 | ND | R, >8 | R, >4 | R, >8 | R, >8 | R, >1 | R, >32 | R, >32 | R, >16/4 | R, >1 | R, >2 | S, 1 | S, 0.50 | R, >64 | R, >4/76 |
| 1961 | 10 | ST512 | Red | S, <=4 | S, 2 | S, 2 | R, >32/2 | R, >8 | R, >16 | ND | R, >8 | R, >4 | R, >8 | R, >8 | R, >1 | R, >32 | R, >32 | R, >16/4 | R, >1 | R, >2 | S, 0.5 | S, 0.25 | R, >64 | R, >4/76 |
| 1987 | 11 | ST258 | Violet | R, >16 | R, >4 | R, >4 | R, >32/2 | R, >8 | R, >16 | ND | R, >8 | R, >4 | R, >8 | R, >8 | R, >1 | R, >32 | R, >32 | R, >16/4 | R, >1 | R, >2 | R, 16 | S, 0.50 | S, 32 | R, >4/76 |
| 1998 | 5 | ST3985 | Sporadic | R, >16 | I, 4 | R, >4 | R, >32/2 | R, >8 | R, >16 | ND | R, >8 | R, >4 | R, >8 | R, >8 | R, >1 | R, >8 | R, >8 | R, >16/4 | R, >1 | R, >2 | S, 1 | I, 2 | S, 32 | R, >4/76 |
| 2003 | 12 | ST258 | Violet | R, >16 | R, >4 | R, >4 | R, >32/2 | R, >8 | R, >16 | ND | R, >8 | R, >4 | R, >8 | R, >8 | R, >1 | R, >32 | R, >32 | R, >16/4 | R, >1 | R, >2 | R, 16 | S, 0.75 | S, 32 | R, >4/76 |
| 2018 | 12 | ST258 | Violet | R, >16 | R, >4 | R, >4 | R, >32/2 | R, >8 | R, >16 | ND | R, >8 | R, >4 | R, >8 | R, >8 | R, >1 | R, >32 | R, >32 | R, >16/4 | R, >1 | R, >2 | R, 32 | S, 0.75 | R, >64 | R, >4/76 |
| 2066 | 13 | ST258 | Violet | R, >16 | R, >4 | R, >4 | R, >32/2 | R, >8 | R, >16 | ND | R, >8 | R, >4 | R, >8 | R, >8 | R, >1 | ND | ND | R, >16/4 | R, >1 | R, >2 | R, 32 | ND | S, 32 | R, >4/76 |
| 2079 | 16 | ST258 | Violet | R, >16 | R, >4 | R, >4 | R, >32/2 | R, >8 | R, >16 | ND | R, >8 | R, >4 | R, >8 | R, >8 | R, >1 | R, >8 | R, >8 | R, >16/4 | R, >1 | R, >2 | R, 8 | S, 1 | R, 64 | R, >4/76 |
| 2106 | 5 | ST512 | Green | R, >16 | I, 4 | R, >4 | R, >32/2 | R, >8 | R, >16 | ND | R, >8 | R, >4 | R, >8 | R, >8 | R, >1 | R, >8 | R, >8 | R, >16/4 | R, >1 | R, >2 | S, 1 | I, 2 | R, >64 | R, >4/76 |
| 2110 | 14 | ST258 | Violet | R, >16 | R, 128 | R, >4 | R, >32/2 | R, >8 | R, >16 | ND | R, >8 | R, >4 | R, >8 | R, >8 | R, >1 | R, >32 | R, >32 | R, >16/4 | R, >1 | R, >2 | R, 16 | S, 0.50 | S, 32 | R, >4/76 |
| 2133 | 17 | ST258 | Violet | R, >16 | R, >256 | R, >4 | R, >32/2 | R, >8 | R, >16 | ND | R, >8 | R, >4 | R, >8 | R, >8 | R, >1 | R, >32 | R, >32 | R, >16/4 | R, >1 | R, >2 | R, 8 | S, 0.50 | R, >64 | R, >4/76 |
| 2137 | 16 | ST258 | Violet | R, >16 | R, 4 | R, >4 | R, >32/2 | R, >8 | R, >16 | ND | R, >8 | R, >4 | R, >8 | R, >8 | R, >1 | R, >32 | R, >32 | R, >16/4 | R, >1 | R, >2 | R, 4 | S, <=0.50 | S, 32 | R, >4/76 |
| 2165 | 19 | ST258 | Violet | R, >16 | R, 4 | R, >4 | R, >32/2 | R, >8 | R, >16 | ND | ND | R, >4 | R, >8 | R, >8 | R, >1 | R, >32 | R, >32 | R, >16/4 | R, >1 | R, >2 | R, 4 | S, 0.50 | S, 32 | R, >4/76 |
| 2174 | 19 | ST258 | Violet | R, >16 | R, >256 | R, >4 | R, >32/2 | R, >8 | R, >16 | ND | R, >8 | R, >4 | R, >8 | R, >8 | R, >1 | R, >8 | R, >8 | R, >16/4 | R, >1 | R, >2 | R, 64 | S, 0.50 | R, >64 | R, >4/76 |
| 2176 | 20 | ST258 | Violet | R, >16 | R, >4 | R, >4 | R, >32/2 | R, >8 | R, >16 | ND | R, >8 | R, >4 | R, >8 | R, >8 | R, >1 | R, >8 | R, >8 | R, >16/4 | R, >1 | R, >2 | R, 32 | I, 2 | S, 32 | R, >4/76 |
| 2182 | 18 | ST258 | Violet | R, >16 | R, >4 | R, >4 | R, >32/2 | R, >8 | R, >16 | ND | R, >8 | R, >4 | R, >8 | R, >8 | R, >1 | R, >8 | R, >8 | R, >16/4 | R, >1 | R, >2 | R, 16 | S, 0.50 | R, >64 | R, >4/76 |
| 2183 | 15 | ST258 | Violet | R, >16 | R, >4 | R, >4 | R, >32/2 | R, >8 | R, >16 | ND | R, >8 | R, >4 | R, >8 | R, >8 | R, >1 | R, >8 | R, >8 | R, >16/4 | R, >1 | R, >2 | R, 8 | S, 0.50 | R, 64 | R, >4/76 |
| 2186 | 16 | ST258 | Violet | R, >16 | R, >4 | R, >4 | R, >32/2 | R, >8 | R, >16 | ND | R, >8 | R, >4 | R, >8 | R, >8 | R, >1 | R, >8 | R, >8 | R, >16/4 | R, >1 | R, >2 | R, >64 | S, 1 | R, >64 | R, >4/76 |
| 2205 | 22 | ST512 | Sporadic | R, >16 | I, 4 | R, >4 | R, >32/2 | R, >8 | R, >16 | ND | R, >8 | R, >4 | R, >8 | R, >8 | R, >1 | R, >8 | R, >8 | R, >16/4 | R, >1 | R, >2 | S, 0.5 | S, 1 | S, 32 | R, <=1/19 |
| 2218 | 23 | ST258 | Violet | R, >16 | R, 256 | R, >4 | R, >32/2 | R, >8 | R, >16 | ND | R, >8 | R, >4 | R, >8 | R, >8 | R, >1 | R, >8 | R, >8 | R, >16/4 | R, >1 | R, >2 | R, 8 | S, 1 | R, >64 | R, >4/76 |
| 2221 | 21 | ST258 | Violet | R, >16 | R, >4 | R, >4 | R, >32/2 | R, >8 | R, >16 | ND | R, >8 | R, >4 | R, >8 | R, >8 | R, >1 | R, >32 | R, >32 | R, >16/4 | R, >1 | R, >2 | R, 8 | S, 0.75 | R, >64 | R, >4/76 |
| 2228 | 21 | ST258 | Violet | R, >16 | R, 256 | R, >4 | R, >32/2 | R, >8 | R, >16 | ND | R, >8 | R, >4 | R, >8 | R, >8 | R, >1 | R, >32 | R, >32 | R, >16/4 | R, >1 | R, >2 | R, 8 | S, 1 | R, >64 | R, >4/76 |
