# Supplementary material for: Multiple Klebsiella pneumoniae KPC Clones Contribute to an Extended Hospital Outbreak
Source: Front Microbiol. 2019 Nov 29;10:2767. doi: 10.3389/fmicb.2019.02767 (PMC6896718; doi:10.3389/fmicb.2019.02767)
Supplement: TABLE S2 — Characteristics of the sequenced genomes. [file Table_2.DOCX]

Supplementary Table 2. Characteristics of the sequenced genomes. The following parameters are reported: Number of trimmed Illumina reads used for the assembly, Total genome length (sum of the lengths of the contigs), N Contigs (number of contigs), N50 (length of the shortest contig that, added to the contigs of higher length, represents at least 50% of the assembly), N50n (number of large contigs comprising at least 50% of the assembly), Coverage (total length/total trimmed read length) for the 32 *Klebsiella pneumoniae* KPC strains.

| **Genome ID** | **Patient number** | **ST** | **Cluster** | **Total length** | **N Reads** | **N Contigs** | **N50** | **N50n** | **Coverage** |
| --- | --- | --- | --- | --- | --- | --- | --- | --- | --- |
| 1753 | 1 | ST512 | Sporadic | 5513969 | 6122128 | 117 | 202047 | 9 | 53 |
| 1758 | 2 | ST258 | Sporadic | 5636994 | 4076632 | 144 | 197968 | 9 | 35 |
| 1760 | 4 | ST258 | Sporadic | 5705533 | 7790360 | 204 | 270452 | 8 | 67 |
| 1826 | 3 | ST45 | Sporadic | 5866511 | 7775240 | 236 | 182804 | 11 | 44 |
| 1845 | 5 | ST512 | Green | 5755385 | 11290904 | 234 | 203931 | 10 | 62 |
| 1870 | 5 | ST512 | Green | 5681982 | 7966568 | 258 | 140567 | 13 | 42 |
| 1873 | 5 | ST512 | Green | 5752942 | 6854960 | 227 | 202463 | 10 | 40 |
| 1880 | 6 | ST512 | Green | 5763191 | 15563720 | 256 | 153922 | 10 | 83 |
| 1897 | 7 | ST512 | Red | 5744125 | 6566456 | 215 | 270452 | 8 | 55 |
| 1935 | 8 | ST512 | Red | 5738835 | 4927216 | 219 | 192161 | 11 | 41 |
| 1955 | 9 | ST512 | Red | 5735693 | 10332072 | 186 | 237759 | 9 | 79 |
| 1961 | 10 | ST512 | Red | 5737984 | 12187416 | 195 | 270452 | 8 | 91 |
| 1987 | 11 | ST258 | Violet | 5653341 | 10199520 | 214 | 148646 | 12 | 50 |
| 1998 | 5 | ST3985 | Sporadic | 5519323 | 3337624 | 213 | 174674 | 10 | 28 |
| 2003 | 12 | ST258 | Violet | 5653322 | 3637104 | 187 | 202043 | 10 | 35 |
| 2018 | 12 | ST258 | Violet | 5652311 | 12268440 | 208 | 136854 | 12 | 61 |
| 2066 | 13 | ST258 | Violet | 5681000 | 6040576 | 209 | 202058 | 10 | 58 |
| 2079 | 16 | ST258 | Violet | 5652779 | 4302944 | 183 | 231105 | 9 | 41 |
| 2106 | 5 | ST512 | Green | 5623034 | 3904200 | 245 | 154406 | 13 | 30 |
| 2110 | 14 | ST258 | Violet | 5650346 | 4290016 | 204 | 157686 | 11 | 35 |
| 2133 | 17 | ST258 | Violet | 5649223 | 5117424 | 187 | 203931 | 10 | 45 |
| 2137 | 16 | ST258 | Violet | 5652369 | 6251440 | 253 | 100458 | 18 | 31 |
| 2165 | 19 | ST258 | Violet | 5651515 | 4646488 | 174 | 176879 | 11 | 44 |
| 2174 | 19 | ST258 | Violet | 5653031 | 11375248 | 212 | 203931 | 10 | 59 |
| 2176 | 20 | ST258 | Violet | 5649091 | 4108664 | 174 | 202452 | 10 | 39 |
| 2182 | 18 | ST258 | Violet | 5648496 | 6657032 | 169 | 186863 | 10 | 61 |
| 2183 | 15 | ST258 | Violet | 5689736 | 10992336 | 257 | 213038 | 9 | 81 |
| 2186 | 16 | ST258 | Violet | 5646781 | 16324168 | 158 | 186214 | 10 | 129 |
| 2205 | 22 | ST512 | Sporadic | 5583886 | 6853224 | 166 | 166799 | 12 | 53 |
| 2218 | 23 | ST258 | Violet | 5646182 | 14613288 | 167 | 203931 | 10 | 121 |
| 2221 | 21 | ST258 | Violet | 5649160 | 16177688 | 179 | 202549 | 10 | 119 |
| 2228 | 21 | ST258 | Violet | 5653856 | 10540288 | 215 | 136029 | 14 | 52 |
