# Supplementary material for: Multiple Klebsiella pneumoniae KPC Clones Contribute to an Extended Hospital Outbreak
Source: Front Microbiol. 2019 Nov 29;10:2767. doi: 10.3389/fmicb.2019.02767 (PMC6896718; doi:10.3389/fmicb.2019.02767)
Supplement: TABLE S3 — Predicted antimicrobial susceptibility profiles determined using the Kleborate tool on 32 Klebsiella pneumoniae KPC isolates. [file Table_3.DOCX]

Supplementary Table 3. Predicted antimicrobial susceptibility profiles determined using the Kleborate tool on 32 *Klebsiella pneumoniae* KPC isolates. The software tests 18 common antimicrobial agents, in the table are reported the positive ones: aminoglycosides (Agly), beta-lactamases (Bla), broad spectrum beta-lactamases (Bla_broad), broad spectrum beta-lactamases with resistance to beta-lactamase inhibitors (Bla_broad_inhR), carbapenemase (Bla_Carb), extended spectrum beta-lactamases (Bla_ESBL), colistin (Col), fluoroquinolones (Flq), macrolides (MLS), phenicols (Phe), sulfonamides (Sul), tetracyclines (Tet), trimethoprim (Tmt). The resistance score is also reported in the table, ranging from 0 to 3: 0 = no ESBL, no carbapenemase (regardless of colistin resistance), 1 = ESBL, no carbapenemase (regardless of colistin resistance), 2 = Carbapenemase without colistin resistance (regardless of ESBL), 3 = Carbapenemase with colistin resistance (regardless of ESBL). Following the Kleborate output, imprecise allele matches are indicated with *. Partial matches (the length of match is less than the length of the reported allele) are indicated with ?

| **Genome ID** | **Cluster** | **ST** | **Resistance score** | **N Resistance classes** | **N Resistance genes** | **Aminoglycosides** | **Colistin** | **Fluorquinolones** | **MLS** | **Phe** | **Sul** | **Tet** | **Tmt** | **Bla** | **Bla Carb** | **Bla ESBL** | **Bla broad** | **Bla broad inhR** |
| --- | --- | --- | --- | --- | --- | --- | --- | --- | --- | --- | --- | --- | --- | --- | --- | --- | --- | --- |
| 1753 | Sporadic | ST512 | 3 | 8 | 10 | Aac6-Ib;AadA2 | MgrB-66%;PmrB-64% | ParC-80I;GyrA-83I | - | - | SulI | - | - | AmpH*;OXA-9* | KPC-3 | - | SHV-11 | TEM-122*? |
| 1758 | Sporadic | ST258 | 2 | 6 | 10 | AadB;Aph3-Ia*;Aac6-Ib | - | QnrB5*;ParC-80I;GyrA-83I | - | - | - | - | - | AmpH* | KPC-2 | SHV-12* | - | TEM-54* |
| 1760 | Sporadic | ST258 | 3 | 11 | 14 | AadA2*;Aac6-Ib;Aph3-Ia* | MgrB-66% | ParC-80I;GyrA-83I | MphA | CatA1* | SulI | - | DfrA12 | AmpH*;OXA-9* | KPC-2 | SHV-12 | - | TEM-54* |
| 1826 | Sporadic | ST45 | 2 | 10 | 16 | Aph2-Ib*;Aac6-Im;StrB;StrA*;Aac3-IIa* | - | QnrB1? | - | CatB4 | SulII | TetA | DfrA14 | AmpH*;OXA-1;OXA-9* | KPC-2 | CTX-M-15 | SHV-1? | - |
| 1845 | Green | ST512 | 2 | 10 | 13 | Aph3-Ia*;Aac6-Ib | - | ParC-80I;GyrA-83I | MphA | CatA1* | SulI | - | DfrA12 | AmpH*;OXA-9* | KPC-3 | - | SHV-11 | TEM-54*? |
| 1870 | Green | ST512 | 2 | 10 | 13 | Aph3-Ia*;Aac6-Ib | - | ParC-80I;GyrA-83I | MphA | CatA1* | SulI | - | DfrA12 | AmpH*;OXA-9* | KPC-3 | - | SHV-11 | TEM-54*? |
| 1873 | Green | ST512 | 2 | 10 | 13 | Aph3-Ia*;Aac6-Ib | - | ParC-80I;GyrA-83I | MphA | CatA1* | SulI | - | DfrA12 | AmpH*;OXA-9* | KPC-3 | - | SHV-11 | TEM-54*? |
| 1880 | Green | ST512 | 3 | 11 | 13 | Aph3-Ia*;Aac6-Ib | MgrB-66% | ParC-80I;GyrA-83I | MphA | CatA1* | SulI | - | DfrA12 | AmpH*;OXA-9* | KPC-3 | - | SHV-11 | TEM-54*? |
| 1897 | Red | ST512 | 2 | 10 | 12 | Aph3-Ia* | - | ParC-80I;GyrA-83I | MphA | CatA1* | SulI | - | DfrA12 | AmpH*;OXA-9* | KPC-3 | - | SHV-11 | TEM-54* |
| 1935 | Red | ST512 | 2 | 10 | 12 | Aph3-Ia* | - | ParC-80I;GyrA-83I | MphA | CatA1* | SulI | - | DfrA12 | AmpH*;OXA-9* | KPC-3 | - | SHV-11 | TEM-54* |
| 1955 | Red | ST512 | 2 | 10 | 12 | Aph3-Ia* | - | ParC-80I;GyrA-83I | MphA | CatA1* | SulI | - | DfrA12 | AmpH*;OXA-9* | KPC-3 | - | SHV-11 | TEM-54* |
| 1961 | Red | ST512 | 2 | 10 | 12 | Aph3-Ia* | - | ParC-80I;GyrA-83I | MphA | CatA1* | SulI | - | DfrA12 | AmpH*;OXA-9* | KPC-3 | - | SHV-11 | TEM-54* |
| 1987 | Violet | ST258 | 3 | 11 | 14 | Aac3-IIa*;Aph3-Ia*;Aac6-Ib | MgrB-77% | ParC-80I;GyrA-83I | MphA | CatA1* | SulI | - | DfrA12 | AmpH*;OXA-9* | KPC-3 | - | SHV-11 | TEM-54*? |
| 1998 | Sporadic | ST3985 | 2 | 3 | 5 | - | - | - | - | - | - | - | - | SHV-187*;AmpH*;OXA-9* | KPC-3 | - | - | TEM-54* |
| 2003 | Violet | ST258 | 3 | 11 | 14 | Aac3-IIa*;Aph3-Ia*;Aac6-Ib | MgrB-77% | ParC-80I;GyrA-83I | MphA | CatA1* | SulI | - | DfrA12 | AmpH*;OXA-9* | KPC-3 | - | SHV-11 | TEM-54*? |
| 2018 | Violet | ST258 | 3 | 11 | 14 | Aac3-IIa*;Aph3-Ia*;Aac6-Ib | MgrB-77% | ParC-80I;GyrA-83I | MphA | CatA1* | SulI | - | DfrA12 | AmpH*;OXA-9* | KPC-3 | - | SHV-11 | TEM-54*? |
| 2066 | Violet | ST258 | 3 | 11 | 14 | Aac6-Ib;Aac3-IIa*;Aph3-Ia* | MgrB-77% | ParC-80I;GyrA-83I | MphA | CatA1* | SulI | - | DfrA12 | AmpH*;OXA-9* | KPC-3 | - | SHV-11 | TEM-54* |
| 2079 | Violet | ST258 | 3 | 11 | 14 | Aac6-Ib;Aac3-IIa*;Aph3-Ia* | MgrB-77% | ParC-80I;GyrA-83I | MphA | CatA1* | SulI | - | DfrA12 | AmpH*;OXA-9* | KPC-3 | - | SHV-11 | TEM-54*? |
| 2106 | Green | ST512 | 2 | 10 | 13 | AadA2*;Aac6-Ib | - | ParC-80I;GyrA-83I | MphA | CatA1* | SulI | - | DfrA12 | AmpH*;OXA-9* | KPC-3 | - | SHV-11 | TEM-54*? |
| 2110 | Violet | ST258 | 3 | 11 | 15 | Aac3-IIa*;AadA2;Aph3-Ia*;Aac6-Ib | MgrB-77% | ParC-80I;GyrA-83I | MphA | CatA1* | SulI | - | DfrA12 | AmpH*;OXA-9* | KPC-3 | - | SHV-11 | TEM-54*? |
| 2133 | Violet | ST258 | 3 | 11 | 15 | Aac3-IIa*;AadA2;Aph3-Ia*;Aac6-Ib | MgrB-77% | ParC-80I;GyrA-83I | MphA | CatA1* | SulI | - | DfrA12 | AmpH*;OXA-9* | KPC-3 | - | SHV-11 | TEM-54*? |
| 2137 | Violet | ST258 | 3 | 11 | 14 | Aac3-IIa*;Aph3-Ia*;Aac6-Ib | MgrB-77% | ParC-80I;GyrA-83I | MphA | CatA1* | SulI | - | DfrA12 | AmpH*;OXA-9* | KPC-3 | - | SHV-11 | TEM-54*? |
| 2165 | Violet | ST258 | 3 | 11 | 14 | Aac3-IIa*;Aph3-Ia*;Aac6-Ib | MgrB-77% | ParC-80I;GyrA-83I | MphA | CatA1* | SulI | - | DfrA12 | AmpH*;OXA-9* | KPC-3 | - | SHV-11 | TEM-54* |
| 2174 | Violet | ST258 | 3 | 11 | 15 | Aac3-IIa*;AadA2*;Aph3-Ia*;Aac6-Ib | MgrB-77% | ParC-80I;GyrA-83I | MphA | CatA1* | SulI | - | DfrA12 | AmpH*;OXA-9* | KPC-3 | - | SHV-11 | TEM-54*? |
| 2176 | Violet | ST258 | 3 | 11 | 14 | Aac6-Ib;Aac3-IIa*;Aph3-Ia* | MgrB-77% | ParC-80I;GyrA-83I | MphA | CatA1* | SulI | - | DfrA12 | AmpH*;OXA-9* | KPC-3 | - | SHV-11 | TEM-54* |
| 2182 | Violet | ST258 | 3 | 11 | 14 | Aac6-Ib;Aac3-IIa*;Aph3-Ia* | MgrB-77% | ParC-80I;GyrA-83I | MphA | CatA1* | SulI | - | DfrA12 | AmpH*;OXA-9* | KPC-3 | - | SHV-11 | TEM-54* |
| 2183 | Violet | ST258 | 3 | 11 | 14 | Aac6-Ib;Aac3-IIa*;Aph3-Ia* | MgrB-77% | ParC-80I;GyrA-83I | MphA | CatA1* | SulI | - | DfrA12 | AmpH*;OXA-9* | KPC-3 | - | SHV-11 | TEM-54* |
| 2186 | Violet | ST258 | 3 | 11 | 15 | AadA2*;Aac6-Ib;Aac3-IIa*;Aph3-Ia* | MgrB-77% | ParC-80I;GyrA-83I | MphA | CatA1* | SulI | - | DfrA12 | AmpH*;OXA-9* | KPC-3 | - | SHV-11 | TEM-54* |
| 2205 | Sporadic | ST512 | 2 | 6 | 9 | Aac6-Ib;AadA2 | - | ParC-80I;GyrA-83I | - | - | SulI | - | - | AmpH*;OXA-9* | KPC-3 | - | SHV-11 | - |
| 2218 | Violet | ST258 | 3 | 10 | 13 | Aac6-Ib;Aac3-IIa*;Aph3-Ia* | MgrB-77% | ParC-80I;GyrA-83I | MphA | CatA1* | SulI | - | DfrA12 | AmpH*;OXA-9* | KPC-3 | - | SHV-11 | - |
| 2221 | Violet | ST258 | 3 | 11 | 14 | Aac6-Ib;Aac3-IIa*;Aph3-Ia* | MgrB-77% | ParC-80I;GyrA-83I | MphA | CatA1* | SulI | - | DfrA12 | AmpH*;OXA-9* | KPC-3 | - | SHV-11 | TEM-54*? |
| 2228 | Violet | ST258 | 3 | 11 | 14 | Aac3-IIa*;Aph3-Ia*;Aac6-Ib | MgrB-77% | ParC-80I;GyrA-83I | MphA | CatA1* | SulI | - | DfrA12 | AmpH*;OXA-9* | KPC-3 | - | SHV-11 | TEM-54*? |
