# Supplementary material for: Multiple Klebsiella pneumoniae KPC Clones Contribute to an Extended Hospital Outbreak
Source: Front Microbiol. 2019 Nov 29;10:2767. doi: 10.3389/fmicb.2019.02767 (PMC6896718; doi:10.3389/fmicb.2019.02767)
Supplement: TABLE S4 — Factors of the 32 Klebsiella pneumoniae KPC isolates. [file Table_4.DOCX]

Supplementary Table 4. Factors of the 32 *Klebsiella pneumoniae* KPC isolates. The analysis was performed with the Kleborate tool that examines five key virulence loci: siderophores yersiniabactin (*ybt*), aerobactin (*iuc*) and salmochelin (*iro*), the genotoxin colibactin (*clb*) and the hypermucoidy determinant (*rmpA/rmpA2*). the Kleborate virulence score is also reported in the table, ranging from 0 to 5: 0 = no virulence loci, 1= yersiniabactin only, 2 = yersiniabactin and colibactin, or colibactin only, 3 = aerobactin and/or salmochelin only (without yersiniabactin or colibactin), 4 = aerobactin and/or salmochelin with yersiniabactin (without colibactin), 5 = yersiniabactin, colibactin and aerobactin and/or salmochelin.

| **Genome ID** | **ST** | **Virulence score** | **Yersiniabactin** | **YbST** | **wzi** | **K locus** | **ybtS** | **ybtX** | **ybtQ** | **ybtP** | **ybtA** | **irp2** | **irp1** | **ybtU** | **ybtT** | **ybtE** | **fyuA** |
| --- | --- | --- | --- | --- | --- | --- | --- | --- | --- | --- | --- | --- | --- | --- | --- | --- | --- |
| 1753 | ST512 | 0 | - | 0 | wzi154 | KL107 | - |  |  |  |  |  |  |  |  |  |  |
| 1758 | ST258 | 1 | Ybt 13; ICEKp2 | 299 | wzi29 | KL106 | 6 | 4 | 20 | 14 | 1 | 126 | 131 | 2 | 4 | 52 | 2 |
| 1760 | ST258 | 1 | Ybt 13; ICEKp2 | 299 | wzi29 | KL106 | 6 | 4 | 20 | 14 | 1 | 126 | 131 | 2 | 4 | 52 | 2 |
| 1826 | ST45 | 1 | Ybt 10; ICEKp4 | 78 | wzi101 | KL24 | 3 | 12 | 4 | 11 | 3 | 21 | 18 | 3 | 10 | 16 | 2 |
| 1845 | ST512 | 0 | - | 0 | wzi154 | KL107 | - | - | - | - | - | - | - | - | - | - | - |
| 1870 | ST512 | 0 | - | 0 | wzi154 | KL107 | - | - | - | - | - | - | - | - | - | - | - |
| 1873 | ST512 | 0 | - | 0 | wzi154 | KL107 | - | - | - | - | - | - | - | - | - | - | - |
| 1880 | ST512 | 0 | - | 0 | wzi154 | KL107 | - | - | - | - | - | - | - | - | - | - | - |
| 1897 | ST512 | 0 | - | 0 | wzi154 | KL107 | - | - | - | - | - | - | - | - | - | - | - |
| 1935 | ST512 | 0 | - | 0 | wzi154 | KL107 | - | - | - | - | - | - | - | - | - | - | - |
| 1955 | ST512 | 0 | - | 0 | wzi154 | KL107 | - | - | - | - | - | - | - | - | - | - | - |
| 1961 | ST512 | 0 | - | 0 | wzi154 | KL107 | - | - | - | - | - | - | - | - | - | - | - |
| 1987 | ST258 | 0 | - | 0 | wzi154 | KL107 | - | - | - | - | - | - | - | - | - | - | - |
| 1998 | ST3985 | 1 | Ybt 4; plasmid | 29-3LV | 0 | - | 18* | 20 | 27 | 18 | 13 | 41 | 71 | 9 | 8 | 26 | 35 |
| 2003 | ST258 | 0 | - | 0 | wzi154 | KL107 | - | - | - | - | - | - | - | - | - | - | - |
| 2018 | ST258 | 0 | - | 0 | wzi154 | KL107 | - | - | - | - | - | - | - | - | - | - | - |
| 2066 | ST258 | 0 | - | 0 | wzi154 | KL107 | - | - | - | - | - | - | - | - | - | - | - |
| 2079 | ST258 | 0 | - | 0 | wzi154 | KL107 | - | - | - | - | - | - | - | - | - | - | - |
| 2106 | ST512 | 0 | - | 0 | wzi154 | KL107 | - | - | - | - | - | - | - | - | - | - | - |
| 2110 | ST258 | 0 | - | 0 | wzi154 | KL107 | - | - | - | - | - | - | - | - | - | - | - |
| 2133 | ST258 | 0 | - | 0 | wzi154 | KL107 | - | - | - | - | - | - | - | - | - | - | - |
| 2137 | ST258 | 0 | - | 0 | wzi154 | KL107 | - | - | - | - | - | - | - | - | - | - | - |
| 2165 | ST258 | 0 | - | 0 | wzi154 | KL107 | - | - | - | - | - | - | - | - | - | - | - |
| 2174 | ST258 | 0 | - | 0 | wzi154 | KL107 | - | - | - | - | - | - | - | - | - | - | - |
| 2176 | ST258 | 0 | - | 0 | wzi154 | KL107 | - | - | - | - | - | - | - | - | - | - | - |
| 2182 | ST258 | 0 | - | 0 | wzi154 | KL107 | - | - | - | - | - | - | - | - | - | - | - |
| 2183 | ST258 | 0 | - | 0 | wzi154 | KL107 | - | - | - | - | - | - | - | - | - | - | - |
| 2186 | ST258 | 0 | - | 0 | wzi154 | KL107 | - | - | - | - | - | - | - | - | - | - | - |
| 2205 | ST512 | 0 | - | 0 | wzi154 | KL107 | - | - | - | - | - | - | - | - | - | - | - |
| 2218 | ST258 | 0 | - | 0 | wzi154 | KL107 | - | - | - | 4* | - | - | - | - | - | - | - |
| 2221 | ST258 | 0 | - | 0 | wzi154 | KL107 | - | - | - | - | - | - | - | - | - | - | - |
| 2228 | ST258 | 0 | - | 0 | wzi154 | KL107 | - | - | - | - | - | - | - | - | - | - | - |
